# Effect of oxidation at an elevated temperature on the evolution of phases, microstructure, and properties of the oxide films formed on the surface of TiZr

**DOI:** 10.1038/s41598-023-32377-y

**Published:** 2023-03-29

**Authors:** Shih-Hang Chang, Zong-Yu Li

**Affiliations:** Department of Chemical and Materials Engineering, National I-Lan University, I-Lan, 260 Taiwan

**Keywords:** Engineering, Materials science

## Abstract

This study examined the evolution of the microstructure, microhardness, corrosion resistance, and selective leaching properties of oxide films formed on the surface of a Ti–50Zr (%) alloy during heat treatment at 600 °C for various time intervals. According to our experimental results, the growth and evolution of oxide films can be divided into three stages. In stage I (heat treatment for less than 2 min), ZrO_2_ was first formed on the surface of the TiZr alloy, which slightly improved its corrosion resistance. In stage II (heat treatment for 2–10 min), the initially generated ZrO_2_ is gradually transformed into ZrTiO_4_ from the top to the bottom of the surface layer. The formation of ZrTiO_4_ significantly improves the microhardness and corrosion resistance of the alloy. In stage III (heat treatment for more than 10 min), microcracks appeared and propagated on the surface of the ZrTiO_4_ film, deteriorating the surface properties of the alloy. The ZrTiO_4_ began to peel off after heat treatment for more than 60 min. The untreated and heat-treated TiZr alloys exhibited excellent selective leaching properties in Ringer’s solution, whereas a trace amount of suspended ZrTiO_4_ oxide particles formed in the solution after soaking the 60 min heat-treated TiZr alloy for 120 days. Surface modification of the TiZr alloy by generating an intact ZrTiO_4_ oxide film effectively improved its microhardness and corrosion resistance; however, oxidation should be performed appropriately to obtain materials with optimal properties for biomedical applications.

## Introduction

Metallic materials such as stainless steel, cobalt-chromium alloys, pure titanium, Ti–6Al–4V alloys, and shape memory alloys, are normally used as replacements for structural components of the human body that require sufficient strength^1–3^. Compared with stainless steel and cobalt-chromium alloys, titanium alloys, such as commercial purity Ti, α-type Ti, (α + β)-type Ti, and low elastic modulus β-type Ti alloys with non-toxic β-stabilizers have widespread applications in biomedical implants owing to their optimal mechanical properties, good corrosion resistance, excellent biocompatibility, and nontoxicity^4–8^. Ti-based shape memory alloys are widely used in stents, orthodontic wires, and rotary endodontic instruments because of their unique superelasticity and shape-memory effects. In addition to the aforementioned metallic biomaterials, several studies have reported that TiZr alloys are potential metallic biomaterials that are particularly suitable for dental implants because of their good corrosion resistance, mechanical strength, and biocompatibility^9–11^. Chen et al.^12^ demonstrated that apatite-coated TiZr alloys are promising artificial bone substitutes or hard-tissue replacement materials for heavy load-bearing applications. Sista et al.^13^ compared the surface and biological properties of TiZr and TiNb alloys and concluded that TiZr alloys are more suitable as implant materials than TiNb alloys because they possess a better biological profile based on the initial attachment of MC3T3-E1 osteoblast cells.

Akimoto et al.^14^ investigated the corrosion resistance and biocompatibility of TiZr alloys with various Zr proportions using anodic polarization and immersion tests in lactic acid and artificial saliva. They reported that TiZr alloys exhibited better corrosion resistance in clinical applications when the Zr content was less than 50%. Wang et al.^15^ studied the microstructure, mechanical properties, and in vitro biocompatibility of TiZr alloys with various chemical compositions. They reported that the hardness, compressive strength, and bending strength of the TiZr alloys increased with increasing Zr content. In vitro cytotoxicity tests revealed that the TiZr alloys exhibited no cytotoxic effects on MG-63 cells, indicating their great potential for dental applications. Xia et al.^16^ reported that the addition of Ni atoms to TiZr alloys enhanced their mechanical properties while simultaneously reducing their plasticity. They also demonstrated that the corrosion resistance of the alloys was significantly improved by the addition of Ni atoms owing to the stabilizing effect of Ni on the passivation films.

Metallic biomaterials typically exhibit good corrosion resistance; however, long-term interactions between implants and living tissues or body fluids may cause implant degradation, increasing the leaching of potentially toxic metal ions^17,18^. Therefore, to improve their corrosion resistance, biocompatibility, and bioactivity, metallic implant surfaces are typically protected by coating them with ceramics, polymers, or composite materials. Thermal oxidation has also been widely applied to improve the corrosive properties of Ti-based alloys because of the formation of highly passive TiO_2_ oxide films during heat treatment^19–24^. Recently, Cui et al.^25,26^ reported that the formation of dense oxide films on the surface of TiZr-based alloys effectively improved wear resistance in an atmospheric environment and corrosion resistance in a simulated human body or seawater environment. Correa et al.^27^ also concluded that Ti–Zr–Mo alloys are suitable for biomedical implants after thermal oxidation because of the formation of oxide layers on their surfaces. They reported that thermal treatments affected the composition, morphology, roughness, wettability, and microhardness of the Ti–Zr–Mo alloys.

The aforementioned studies have demonstrated that heat treatment can effectively improve the surface properties of TiZr or TiZr-based alloys. However, most of these studies only investigated the properties of TiZr alloys oxidized at specific temperatures and time intervals. In other words, the mechanism of oxide film growth during heat treatment has not been studied in detail. Because the surface properties of TiZr alloys are closely related to the phase, chemical composition, crystal structure, and morphology of the oxide film formed, it is critical to understand the variation in these properties during heat treatment. Accordingly, we could achieve the optimal performance of TiZr alloys by carefully controlling the oxidation processes to obtain an oxide film with the desired surface properties. Therefore, this study aims to investigate the evolution of oxide films formed on the surfaces of TiZr alloys during heating at 600 °C for various time intervals. The effects of heat treatment on the corrosion properties, microhardness, and selective leaching behavior, of the TiZr alloys are also discussed.

## Experimental

### Preparation and heat treatments of TiZr alloy specimens

The Ti–50Zr (wt%) alloy used in this study was prepared from high-purity Ti (99.99 wt%) and Zr (99.9 wt%) using a conventional vacuum arc remelter (VAR). The VAR used in this study was custom-manufactured by Yongfa Co., Taiwan, with a nominal output power of 10 kW. The raw materials were placed in a water-cooled copper crucible in the VAR system. After the furnace was evacuated and purged with high-purity Ar for five cycles, it was filled with high-purity Ar at a pressure of approximately 260 Torr. Subsequently, the raw materials were cast into ingots via arc melting in an Ar atmosphere. The ingot was remelted six times to obtain a homogenized TiZr alloy and then cut into specimens with dimensions of approximately 20.0 mm × 20.0 mm × 3 mm using a low-speed diamond saw (IsoMet LS, Buehler). The surface of each specimen was progressively ground using an abrasive paper and polished with Al_2_O_3_ powder. The TiZr alloy specimens were then heat-treated in a furnace at 600 °C for various time intervals from 1 to 60 min. The high-temperature furnace used in this study was purchased from Yongfa Co., Taiwan (Model: YH-143, operating voltage: 220 V, single phase, frequency: 60 Hz, current: 30 A). The chamber size of the high-temperature furnace is approximately 30.0 mm × 23.0 mm × 21.0 mm. Before heat treatment, the high-temperature furnace was heated from room temperature to 600 °C in an air atmosphere and maintained at this temperature for more than 60 min. Then, the TiZr alloy specimen was placed into the furnace for subsequent heat treatment. After heat treatment, the specimen was removed from the furnace and cooled to room temperature under an ambient atmosphere.

### XRD and GIXRD measurements

The crystallographic structure of the heat-treated TiZr alloy was determined using X-ray diffraction (XRD; Bruker D2 PHASER XE-T) with Cu Kα radiation (λ = 0.154 nm) at room temperature. The crystallographic structures of the oxide films on the surface of the heat-treated TiZr alloy were analyzed by grazing incidence XRD (GIXRD; Bruker D8 DISCOVER SSS) with Cu Kα radiation (λ = 0.154 nm) at room temperature.

### SEM and EDS measurements

Surface morphologies and cross-sectional images of the heat-treated TiZr alloy were obtained using field-emission scanning electron microscopy (FESEM; Thermo Scientific Phenom ProX G6). The chemical composition of the heat-treated TiZr alloy was determined using energy-dispersive spectroscopy (EDS; Oxford Instruments X-act 10 mm^2^ SDD Detector).

### Electrochemical tests

In the electrochemical tests, the cathodic and anodic polarization Tafel curves of each specimen were obtained using a WonATech ZIVE SP1 electrochemical workstation. The counter and reference electrodes were a platinum plate and a saturated calomel electrode (SCE), respectively. Ringer’s solution, which is an isotonic solution with a composition similar to that of bodily fluids, was used as the test solution. The average corrosion potential (*E*_corr_), average corrosion current density (*i*_corr_), and average polarization resistance (*R*_p_) of each specimen were calculated from seven Tafel curves, and the maximum and minimum values were removed.

### Microhardness tests

The microhardness values of the heat-treated TiZr alloy specimens were determined using a Wilson Instruments 402 MVD Vickers hardness tester at a load of 300 g and a dwell time of 10 s. Microhardness was calculated as the average of seven data points, with the maximum and minimum values omitted.

### Selective leaching behaviors

For the selective leaching measurements, the heat-treated TiZr alloy specimens were individually immersed in test flasks containing 500 mL of Ringer’s solution. Each test flask was incubated at 37 °C in an orbital shaker for 120 d. The metal ion concentrations in the Ringer’s solution after leaching were determined using inductively coupled plasma mass spectrometry (ICP-MS; Agilent 7500ce).

## Results

### XRD and GIXRD results for the heat-treated TiZr alloy

**Figure 1:**
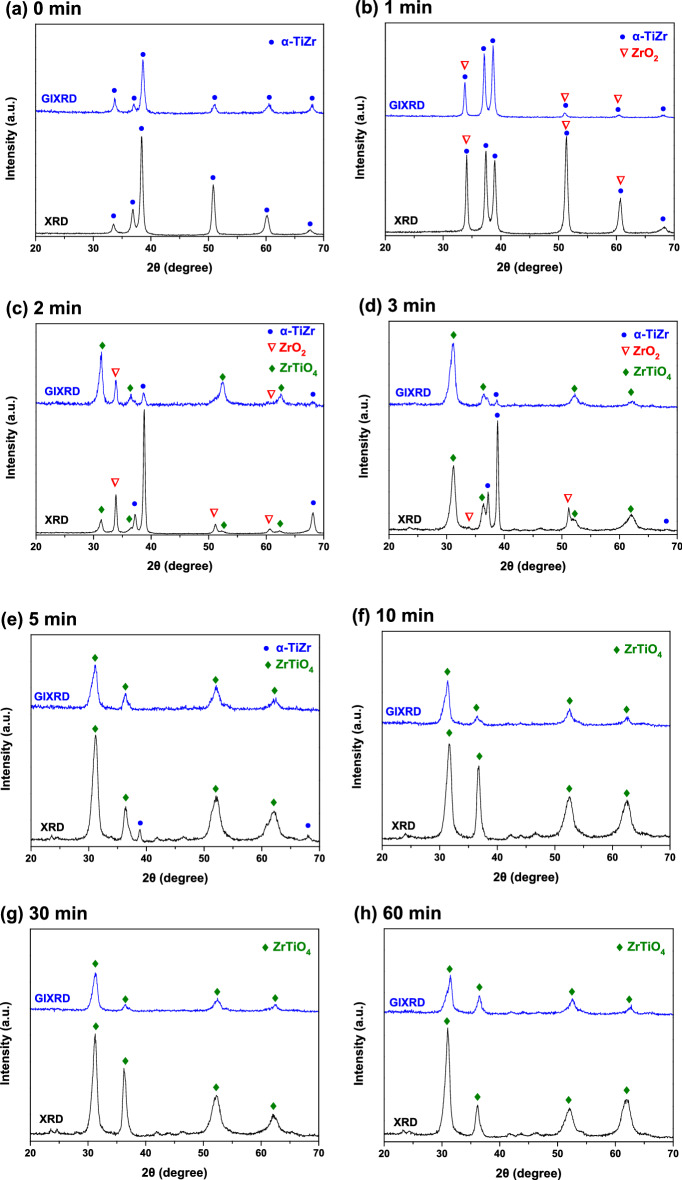
XRD and GIXRD patterns of the TiZr alloy specimens heat-treated at 600 °C for (**a**) 0 min, (**b**) 1 min, (**c**) 2 min, (**d**) 3 min, (**e**) 5 min, (**f**) 10 min, (**g**) 30 min, and (**h**) 60 min.

Figure 1a–h show the XRD patterns of the TiZr alloy specimens heat-treated at 600 °C for 0, 1, 2, 3, 5, 10, 30, and 60 min, respectively. As shown in Fig. 1a, the untreated TiZr alloy exhibits diffraction peaks at approximately 2θ = 33.5, 36.8, 38.4, 50.8, 60.1, and 67.6°, corresponding to the (100), (002), (101), (102), (110), and (103) diffraction planes of α-TiZr, respectively. Figure 1b shows that the XRD patterns of the TiZr alloy heat-treated for 1 min were similar to those of the untreated TiZr alloy. However, the relative intensities of the diffraction peaks of the TiZr alloy heat-treated for 1 min were not identical to those of the untreated TiZr alloy. This was because ZrO_2_ formed on the surface of the TiZr alloy heat-treated for 1 min^25^. The diffraction peaks of the (002), (220), and (311) diffraction planes of ZrO_2_ oxide are located at approximately 2θ = 33.5, 50.8, and 60.1°, respectively. These diffraction peaks nearly coincide with those of α-TiZr and were difficult to distinguish clearly.

As shown in Fig. 1c, in addition to the diffraction peaks of α-TiZr and ZrO_2_, the TiZr alloy heat-treated for 2 min exhibits additional diffraction peaks at 2θ = 31.4°, 36.4°, 52.0°, and 62.1°, which were ascribed to the (111), (002), (220), and (311) diffraction planes of ZrTiO_4_^26^. Figure 3d shows that the diffraction patterns of the TiZr alloy heat treated for 3 min are similar to those of the TiZr alloy heat treated for 2 min, whereas the diffraction peaks of ZrTiO_4_ have higher intensities than those of ZrO_2_. The diffraction peaks of the TiZr alloy heat-treated for 5 min (Fig. 1e) were primarily attributed to ZrTiO_4_, the diffraction peaks of α-TiZr and ZrO_2_ have extremely weak intensities or are undetectable. Figure 1f–h show that only the diffraction peaks of ZrTiO_4_ were observed in the TiZr alloy heat-treated at 600 °C for more than 10 min.

To identify the crystallographic structures of the oxide films formed on the shallow surfaces of the heat-treated TiZr alloy specimens, specimens identical to those used in the XRD measurements were analyzed using GIXRD. The GIXRD patterns are also presented in Fig. 1 for comparison. Figure 1a,b show that the GIXRD patterns of the untreated TiZr alloy and the TiZr alloy heat-treated for 1 min are very similar to the XRD results. This indicates that the crystal structures of the shallow and deep surface layers were almost identical in the untreated TiZr alloy and TiZr alloy heat-treated for 1 min. As shown in Fig. 1c, the GIXRD patterns of the TiZr alloy heat-treated for 3 min were similar to the XRD results of the identical specimen, whereas the intensities of the diffraction peaks of ZrTiO_4_ in the GIXRD results were more significant than those in the XRD results. This suggested that the preliminarily generated ZrTiO_4_ was primarily located on the superficial surface of the oxide film. Figure 1d shows that the diffraction peaks of the TiZr alloy heat-treated for 3 min were quite different from the XRD results. The pattern of the TiZr alloy heat-treated for 3 min mainly consists of ZrTiO_4_ peaks, with only a weak diffraction peak of α-TiZr. This indicates that ZrTiO_4_ became progressively thicker with increasing heat treatment time. In addition, as shown in Fig. 1e–h, only the diffraction peaks of ZrTiO_4_ are detected in the GIXRD patterns of the TiZr alloy heat-treated for more than 5 min.

### SEM images and EDS analysis of the heat-treated TiZr alloy

**Figure 2:**
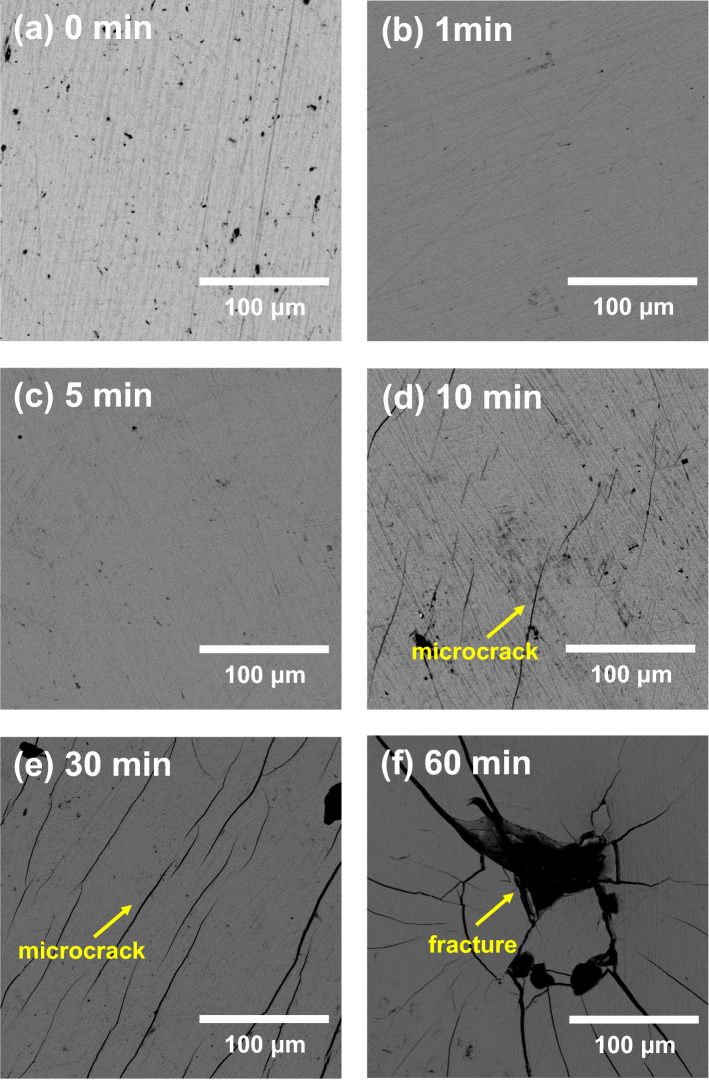
Top view SEM images of the TiZr alloy specimens heat-treated at 600 °C for (**a**) 0 min, (**b**) 1 min, (**c**) 5 min, (**d**) 10 min, (**e**) 30 min, and (**f**) 60 min.

Figure 2a–f present top-view SEM images of the TiZr alloy specimens heat-treated at 600 °C for 0, 1, 5, 10, 30, and 60 min, respectively. The untreated TiZr alloy and the TiZr alloy heat-treated for 1 and 5 min (Fig. 2a–c) exhibit intact and flat surfaces, with only minor scratches. However, as shown in Fig. 2d, microcracks are already observed on the surface of the TiZr alloy heat-treated for 10 min. More cracks appeared and propagated on the surface of the TiZr alloy heat-treated for 30 min (Fig. 2e). After the heat treatment for 60 min (Fig. 2f), the oxide films on the surface of the TiZr alloy are severely fractured and begin to peel off.

**Figure 3:**
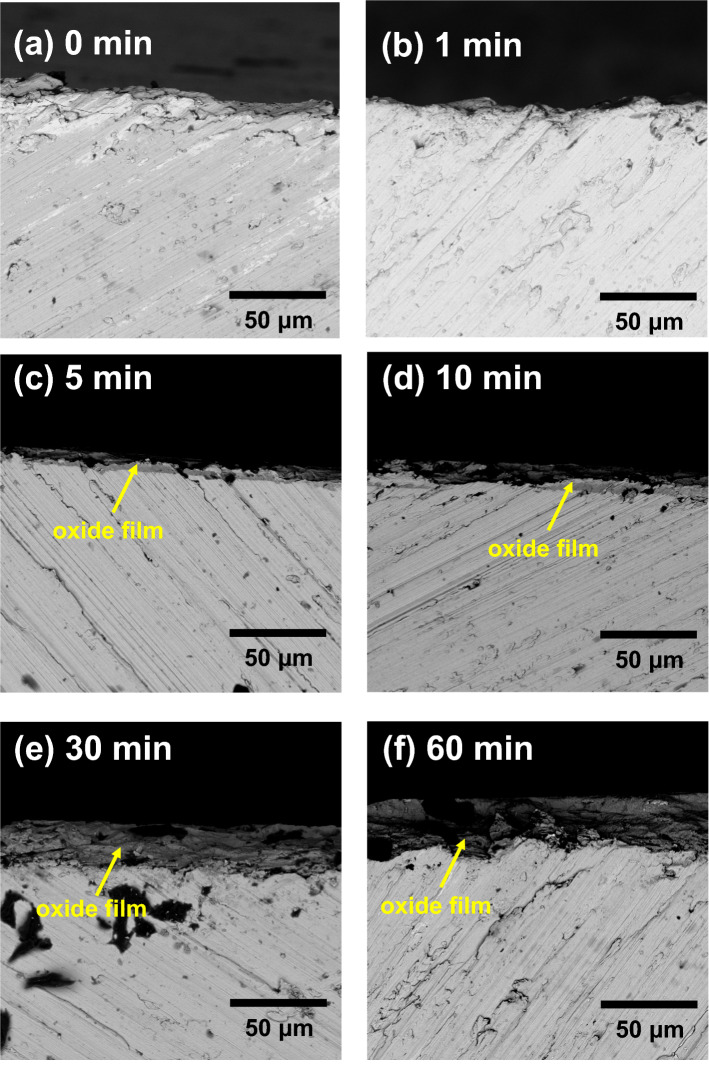
Cross-sectional view SEM images of the TiZr alloy specimens heat-treated at 600 °C for (**a**) 0 min, (**b**) 1 min, (**c**) 5 min, (**d**) 10 min, (**e**) 30 min, and (**f**) 60 min.

**Figure 4:**
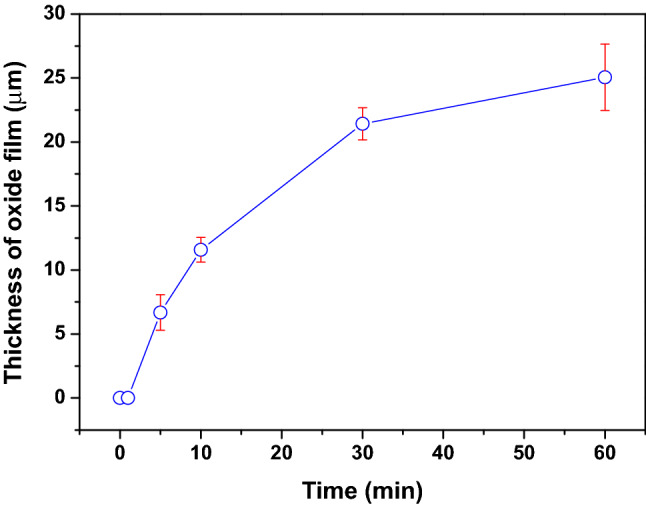
Thickness of the oxide film formed on the surface of the TiZr alloy as a function of heat treatment time.

Figure 3a–f show cross-sectional SEM images of the TiZr alloy heat-treated at 600 °C for 0, 1, 5, 10, 30, and 60 min, respectively. In Fig. 3a,b, no significant oxidation film was observed on the surface of the untreated TiZr alloy and the TiZr alloy heat-treated for 1 min. As shown in Fig. 3c, a thin oxide film with a thickness of approximately 7 μm is observed on the surface of the TiZr alloy after heat treatment for 5 min. As shown in Fig. 3d–f, the thickness of the oxide films gradually increased from approximately 11–25 μm with the extension of the heat treatment time from 10 to 60 min. Figure 4 shows the thickness of the oxide films determined from Fig. 3 as a function of heat-treatment time. Figure 4 shows that the growth rate of the oxide film on the surface of the TiZr alloy is approximately 1 μm/min in the first 10 min and gradually decreases after that. This is because the oxide film initially formed on the surface inhibited further oxidation of the alloy.

Table 1 lists the EDS results for the untreated and heat-treated TiZr alloys. As shown in Table 1, the chemical composition of the untreated TiZr alloy was approximately Ti-51.79Zr. The oxygen content in the untreated TiZr alloy was extremely low, indicating no oxide films formed on its surface. The oxygen weight percentage increased to 5.74% after the TiZr alloy was heat-treated at 600 °C for 1 min, suggesting that an oxide film was generated at this stage. The oxygen content was approximately 37% after the TiZr alloy was heat-treated at 600 °C for 5 min, indicating that the formed oxide film grew at this stage. The oxygen content did not change significantly when the TiZr alloys were heat-treated at 600 °C for more than 5 min, even though the thickness of the oxide film progressively increased with the heat treatment duration, as illustrated in Fig. 3. This was because the oxide films formed on these specimens were thicker than the sampling depth of EDS.

**Table 1:** EDS analysis of the untreated and heat-treated TiZr alloy specimens.

Samples (min)	Ti (wt%)	Zr (wt%)	O (wt%)
0	48.21	51.79	Undetectable
1	45.59	48.67	5.74
5	30.17	32.51	37.33
10	31.06	34.02	34.92
30	30.36	32.98	36.65
60	29.44	33.31	37.25

### Electrochemical results for the heat-treated TiZr alloy

**Figure 5:**
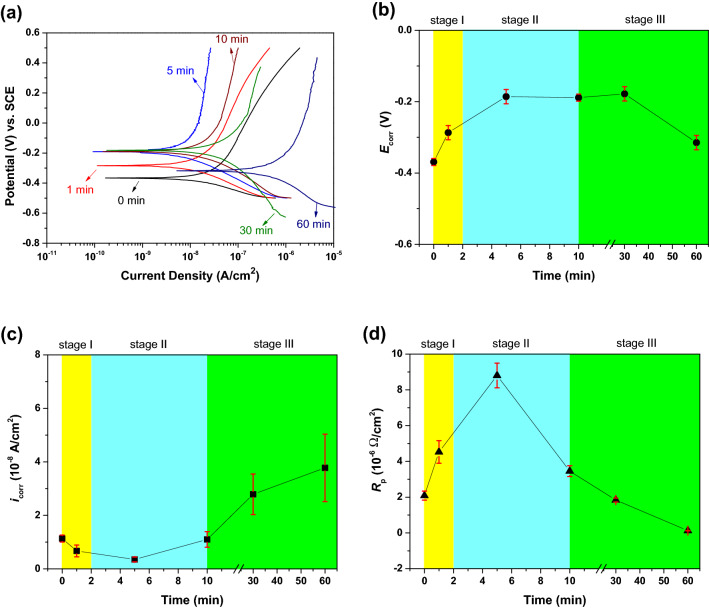
(**a**) Cathodic and anodic polarization Tafel curves for the TiZr alloy specimens heat-treated at 600 °C for various time intervals. The average (**b**) *E*_corr_, (**c**) *i*_corr_, and (**d**) *R*_p_ values of the TiZr alloy as a function of heat treatment time.

Figure 5a shows the selected cathodic and anodic polarization Tafel curves for the TiZr alloy heat-treated at 600 °C for various time intervals. Seven Tafel curves were obtained for each specimen to determine the average *E*_corr_, *i*_corr_, and *R*_p_ values; only one Tafel curve for each specimen is presented in Fig. 5a for clarity. Table 2 lists the average *E*_corr_, *i*_corr_, and *R*_p_ values of untreated and heat-treated TiZr alloys. The average *E*_corr_, *i*_corr_, and *R*_p_ values of the untreated and heat-treated TiZr alloys as a function of heat-treatment time are plotted in Fig. 5b–d.

**Table 2:** The average *E*_corr_, *i*_corr_, and *R*_p_ values of the untreated and heat-treated TiZr alloy specimens.

Sample (min)	*E*_corr_ (V)	*i*_corr_ (A/cm^2^)	*R*_p_ (Ω/cm^2^)
0	− 0.37 ± 0.01	(1.13 ± 0.13) × 10^−8^	(2.09 ± 0.25) × 10^−6^
1	− 0.29 ± 0.02	(0.67 ± 0.22) × 10^−8^	(4.53 ± 0.63) × 10^−6^
5	− 0.19 ± 0.02	(0.35 ± 0.09) × 10^−8^	(8.80 ± 0.69) × 10^−6^
10	− 0.19 ± 0.01	(1.10 ± 0.29) × 10^−8^	(3.46 ± 0.30) × 10^−6^
30	− 0.18 ± 0.02	(2.79 ± 0.76) × 10^−8^	(1.84 ± 0.11) × 10^−6^
60	− 0.31 ± 0.02	(3.77 ± 1.26) × 10^−8^	(0.13 ± 0.01) × 10^−6^

According to Fig. 5b, the average *E*_corr_ value of the untreated TiZr alloy is − 0.37 ± 0.01 V. The *E*_corr_ values of the heat-treated TiZr alloy gradually increase from − 0.29 ± 0.02 to − 0.18 ± 0.02 V with the increase in the heat treatment time from 1 to 30 min. Nevertheless, the TiZr alloy heat-treated for 60 min exhibits a low* E*_corr_ value of only − 0.31 ± 0.02 V. As shown in Fig. 5c, the average *i*_corr_ value of the untreated TiZr alloy is (1.13 ± 0.13) × 10^–8^ A/cm^2^. Initially, the average *i*_corr_ value of the TiZr alloy gradually decreases, reaching a minimum of (0.35 ± 0.09) × 10^–8^ A/cm^2^ for the heat-treatment time of 5 min. However, with further extension of the heat-treatment time, the average *i*_corr_ value of the TiZr alloy gradually increased. As shown in Fig. 5d, the average *R*_p_ value of the untreated TiZr alloy is (2.09 ± 0.25) × 10^–6^ Ω/cm^2^. Initially, the average *R*_p_ value of the TiZr alloy gradually increases to a maximum of (8.80 ± 0.69) × 10^–6^ Ω/cm^2^ at the heat treatment time of 5 min. With further extension of the heat-treatment time, the average *R*_p_ value of the TiZr alloy gradually decreased.

Corrosion potential is a characteristic of a material surface that loses electrons in the presence of an electrolyte. Current density and polarization resistance refer to the degree and speed of corrosion of the material and the resistance of the specimen to oxidation, respectively. As shown in Fig. 5, the corrosion resistance of the TiZr alloy was improved by heating at 600 °C for less than 10 min because the TiZr alloy specimens heat-treated for 10 min or less exhibited lower *E*_corr_ and *i*_corr_ values and a higher *R*_p_ value than the untreated alloy. Simultaneously, heat treatment for more than 10 min reduced the corrosion resistance of the heat-treated TiZr alloys. Nevertheless, the polarization Tafel curves can only specify the general corrosion properties of the samples, and further detailed electrochemical impedance spectroscopy tests are required to investigate the related corrosion properties of the oxide films.

### Microhardness of the heat-treated TiZr alloy

**Figure 6:**
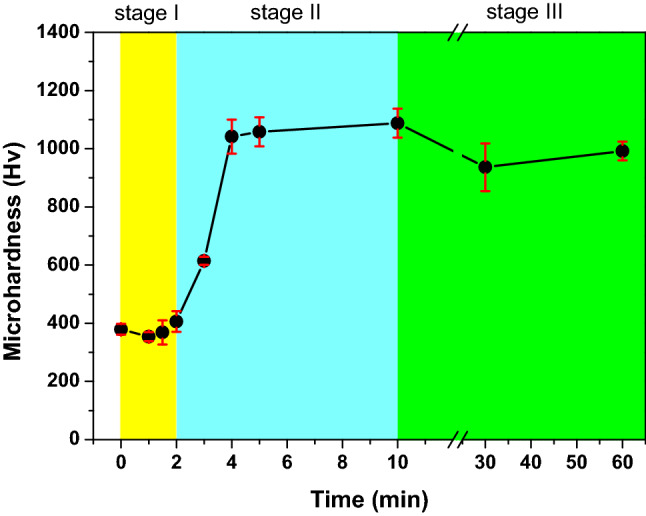
Microhardness of the TiZr alloy specimens as a function of heat treatment time.

Figure 6 plots the measured microhardness of the untreated and heat-treated TiZr alloy as a function of heat treatment time. Figure 6 shows that the untreated TiZr alloy exhibits a low microhardness of only approximately 380 Hv. The microhardness of the TiZr alloy heat-treated for 2 min is slightly higher (approximately 400 Hv). With further extension of the heat treatment time to 3 and 4 min, the microhardness TiZr alloy considerably increases to approximately 615 and 1040 Hv, respectively. The TiZr alloy specimens heat-treated for 5 and 10 min also exhibit microhardness above 1000 Hv. However, with further extension of the heat treatment time to 30 and 60 min, the microhardness of the TiZr alloy slightly decreases to below 1000 Hv.

### Selective leaching behavior of the heat-treated TiZr alloy

For selective leaching measurements, the untreated and heat-treated TiZr alloys were immersed in Ringer’s solution for 120 days to evaluate the leaching rates of Ti and Zr. Notably, the concentrations of Ti and Zr in the Ringer’s solution were both lower than the detection limit of the ICP-MS instrument for each specimen. This indicates that both the untreated and heat-treated TiZr alloys exhibited extremely low leaching rates for both Ti and Zr. The considered TiZr alloy exhibited better selective leaching properties than the Ti- and Cu-based shape memory alloys^28–31^ and high-entropy alloys^32,33^ reported in our previous studies.

## Discussion

**Figure 7:**
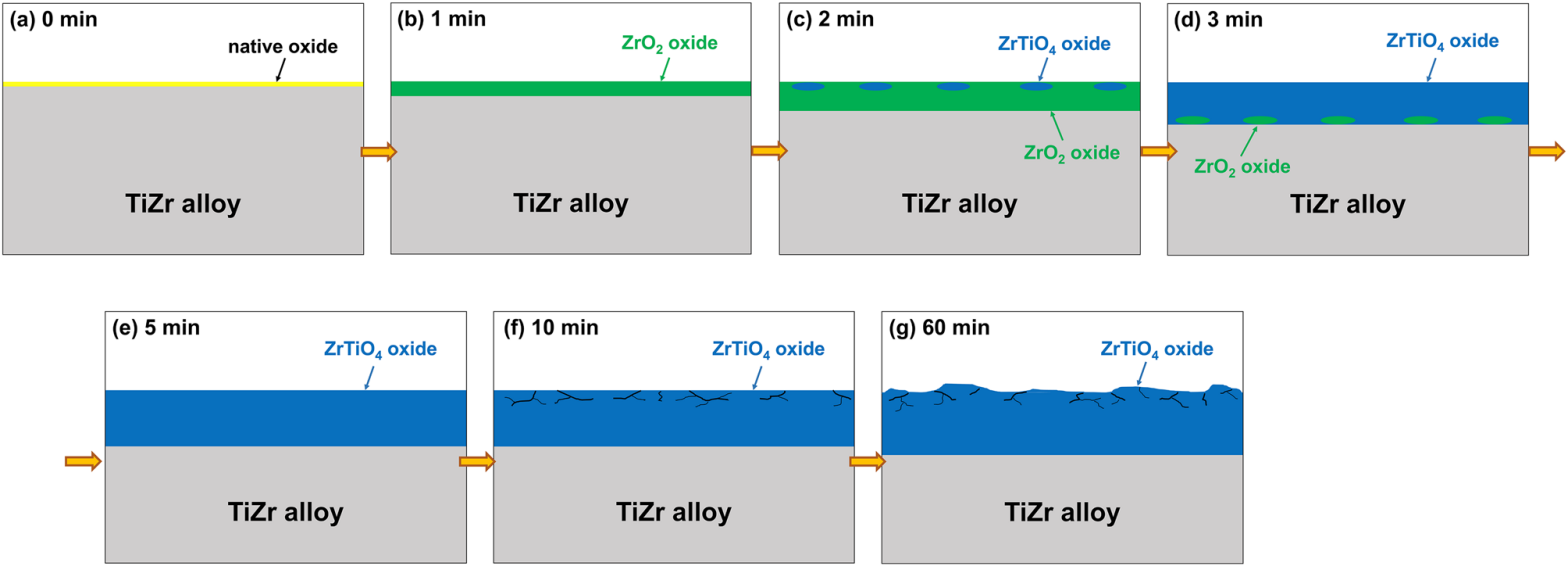
Evolution of the oxide film formed on the surface of the TiZr alloy during the heat treatment at 600 °C for (**a**) 0, (**b**) 1, (**c**) 2, (**d**) 3, (**e**) 5, (**f**) 10, and (**g**) 60 min.

According to the XRD, GIXRD, and SEM results shown in Figs. 1, 2 and 3, the microstructure and surface morphology of the oxide films formed on the surface of the TiZr alloy progressively changed with the heat treatment time. Figure 7a–g schematically illustrate the evolution of the oxide film during heat treatment at 600 °C for 0, 1, 2, 3, 5, 10, and 60 min, respectively. Before oxidation, only native oxide films are present on the surface of the TiZr alloy, as shown in Fig. 7a. Based on the XRD and GIXRD results shown in Fig. 1b, ZrO_2_ was formed on the surface of the TiZr alloy during heat treatment for 1 min, as illustrated in Fig. 7b. As illustrated in Fig. 7c and according to the XRD and GIXRD results (Fig. 1c), the shallow surface layer of the preliminarily formed ZrO_2_ began to transform into ZrTiO_4_ after heat treatment for 2 min.

After heat treatment for 3 min, the diffraction peaks of the ZrO_2_ oxide film and α-TiZr could still be observed in the XRD pattern (Fig. 1d), whereas only an extremely weak α-TiZr diffraction peak could be observed in the GIXRD pattern. This suggests that the oxide films on the surface of the TiZr alloy heat-treated for 3 min primarily consisted of ZrTiO_4_, whereas residual ZrO_2_ only existed deep beneath the ZrTiO_4_ film, as illustrated in Fig. 7d. In the XRD and GIXRD patterns (Fig. 1) of the TiZr alloy heat-treated for more than 5 min, only the diffraction peaks of ZrTiO_4_ oxide were observed. This indicates that the phases and microstructure of the oxide film on the surface of the alloy did not change thereafter, as illustrated in Fig. 7e–g. However, SEM (Fig. 2d) revealed that microcracks appeared and propagated on the surface of ZrTiO_4_ after the heat treatment of the TiZr alloy for 10 min, as illustrated in Fig. 7f. In addition, as shown in Fig. 2f, the surface of the ZrTiO_4_ oxide film began to peel off after heat treatment for more than 60 min, as shown in Fig. 7g.

According to the schematic shown in Fig. 7, the oxidation of the TiZr alloy can be divided into three stages. In stage I, during the first 2 min of heat treatment at 600 °C, the oxidation film consisting primarily of ZrO_2_ is formed on the surface of the TiZr alloy. The diffraction peaks of TiO_2_ oxide were not detected in both the XRD or GIXRD results, indicating that ZrO_2_ was more easily formed on the surface of the TiZr alloy during the heat treatment at 600 °C. This can be explained by the lower Gibbs free energy of formation (ΔG_*f*_) of ZrO_2_ at 600 °C (− 928.46 kJ/mol) compared to TiO_2_ (− 782.97 kJ/mol)^34^. At this stage, only a small amount of ZrO_2_ was transformed into ZrTiO_4_. ZrO_2_ is gradually transformed into ZrTiO_4_ with an increase in the heat-treatment time because the elastic strain energy of the lattice can be reduced by adjusting the crystal structure and lattice constants^26^. As shown in Fig. 5, *i*_corr_ decreased and *R*_p_ increased at this stage, indicating that the heat-treated TiZr alloy exhibited better corrosion resistance than the untreated alloy. However, as shown in Fig. 6, the microhardness of the TiZr alloy at this stage is comparable to that of the untreated TiZr alloy, suggesting that the initially formed ZrO_2_ does not improve the surface hardness of the TiZr alloy. This is because the initially formed ZrO_2_ film was not thick enough to provide sufficient protection during the electrochemical and microhardness tests.

In stage II, during the heat treatment of the TiZr alloy for 2–10 min, most of the initially formed ZrO_2_ was progressively transformed into ZrTiO_4_, and only a small amount of residual ZrO_2_ was present beneath the formed ZrTiO_4_. As shown in Figs. 5 and 6, the heat-treated TiZr alloy exhibits good corrosion resistance and high microhardness during this stage. ZrTiO_4_ oxide provides better protection than ZrO_2_ oxide and therefore improves the electrochemical and mechanical properties of alloys^25,26^. Compared with previous studies^25^, the microhardness of the untreated TiZr alloy used in this study (approximately 380 Hv) was slightly higher than those of the untreated Ti-60Zr (approximately 340 Hv) and Ti-80Zr (approximately 329 Hv) alloys. This may be because the solid-solution hardening of α-phase is more significant in a nearly equal weight ratio TiZr alloy than Ti-60Zr and Ti-80Zr alloys. The microhardness of TiZr alloy could be increased to approximately 1050 Hv after being heat-treated at 600 °C for 5 min, which is much higher than the other untreated TiZr alloys reported before. However, this value was lower than the Ti-60Zr (approximately 1476 Hv) and Ti-80Zr alloys (approximately 1425 Hv) heat-treated at 500 °C for 2 h in air. This difference is not yet clear, possibly because the microstructure and mechanical properties of the ZrTiO_4_ oxide film show some discrepancies for Ti-*x*Zr alloys with different chemical compositions.

In stage III, during heat treatment for more than 10 min, only ZrTiO_4_ remains on the surface of TiZr alloy. Nevertheless, as shown in Figs. 5 and 6, the corrosion resistance and microhardness of the heat-treated TiZr alloy slightly decreased during this stage. These results are attributed to the formation of abundant cracks on the surface of the ZrTiO_4_ film, which deteriorated the electrochemical and mechanical properties of the alloy. Although the electrochemical and mechanical properties of TiZr alloys are still acceptable at this stage, the desquamation of the oxide film is a critical issue when TiZr alloys are considered for biomedical applications. For selective leaching measurements, untreated and heat-treated TiZr alloy specimens were individually immersed in test flasks containing 500 mL of Ringer’s solution for 120 days. Ringer’s solution immersed in the TiZr alloys heat-treated at 600 °C for less than 30 min remained clear and transparent for 120 days.

Nevertheless, a trace amount of white suspended solid particles appeared in Ringer’s solution after soaking the TiZr alloy heat-treated at 600 °C for 60 min for 120 days. Concentrated nitric acid could dissolve these suspended solid particles in Ringer’s solution. The concentrations of Ti and Zr in the obtained solution were 1066 and 1399 ppb, respectively, as detected using ICP-MS. These suspended solid particles were only obtained in Ringer’s solution after soaking the TiZr alloy heat-treated for 60 min; therefore, they should be TiZrO_4_ that peeled off from the surface of the alloy. In addition, the ICP-MS results showed that the suspended solid particles possessed a higher Zr content than Ti. This can be explained by the fact that ZrO_2_ oxide films were formed first during the initial oxidation, resulting in a high Zr content on the outer surface of the successively formed ZrTiO_4_ films. Therefore, our results indicate that the corrosion resistance and microhardness of TiZr alloys can be effectively improved by heat treatment, which should be conducted carefully and appropriately to obtain an intact ZrTiO_4_ film on the alloy surface.

## Conclusions

This study investigated the effects of the microstructure and surface morphology of oxide films formed on the surface of TiZr alloys heat-treated at 600 °C on the microhardness and corrosion properties of the alloys. The following conclusions were drawn.

1. The XRD and GIXRD results showed that during the heat treatment at 600 °C for 1 min, a ZrO_2_ oxide film first formed on the surface of the TiZr alloy. In the TiZr alloy heat-treated for 2 min, some of the formed ZrO_2_ on the surface of the oxide film transformed into ZrTiO_4_. In the TiZr Alloy heat treated for 3 min, most ZrO_2_ transformed into ZrTiO_4_, and only a trace amount of residual ZrO_2_ was present beneath the ZrTiO_4_ film. After the heat treatment of the TiZr alloy for more than 5 min, only ZrTiO_4_ was present on the alloy surface.
2. The SEM images revealed that the initially formed oxide films remained intact during heat treatment for less than 5 min. The oxide films formed on the surface of the TiZr alloy began to split and crack at heat treatment times greater than 10 min.
3. The TiZr alloy with an intact ZrTiO_4_ oxide film on its surface exhibited a higher microhardness and better corrosion resistance than the TiZr alloy with a ZrO_2_ film. At the same time, heat treatment for more than 10 min damaged the ZrTiO_4_ protective layer, reducing the microhardness and corrosion resistance of the alloy.
4. Untreated and heat-treated TiZr alloys exhibited extremely low leaching rates in Ringer’s solution. However, trace amounts of white suspended solid particles peeled off from the TiZr alloy heat-treated for 60 min.

## Data Availability

The datasets used and/or analyzed in the current study are available from the corresponding author upon reasonable request.
